# Supplementary material for: Sorting at embryonic boundaries requires high heterotypic interfacial tension
Source: Nat Commun. 2017 Jul 31;8:157. doi: 10.1038/s41467-017-00146-x (PMC5537356; doi:10.1038/s41467-017-00146-x)
Supplement: Supplementary file 2 — Supplementary Software 1 [file 41467_2017_146_MOESM2_ESM.zip › PottsModel/SrcPottsModel/doc/model/SquareCell.html]

SquareCell


JavaScript is disabled on your browser.


Skip navigation links


- Overview
- Package
- Class
- Use
- Tree
- Deprecated
- Index
- Help

- Prev Class
- Next Class

- Frames
- No Frames

- All Classes

- Summary:
- Nested |
- Field |
- Constr |
- Method

- Detail:
- Field |
- Constr |
- Method


model

## Class SquareCell

- java.lang.Object
- - mvc.AObservable
  - - model.Cell
    - - model.SquareCell

- All Implemented Interfaces:
  :   KnowsConstants, IObservable

  ---

    

  ```
  public class SquareCell
  extends Cell
  ```

- - ### Nested Class Summary

    - ### Nested classes/interfaces inherited from class model.Cell

      `Cell.CellType, Cell.Energy, Cell.State`
  - ### Field Summary

    - ### Fields inherited from class model.Cell

      `aConstants, cAreaUnit, cEdgeUnit, cNotificationSource`
  - ### Constructor Summary

    Constructors

    Constructor and Description

    `SquareCell(Cell.CellType pType,
    int pID,
    Lattice pLattice)`

    `SquareCell(int pID,
    Lattice pLattice)`

    `SquareCell(SquareCell pCell)`
  - ### Method Summary

    - ### Methods inherited from class model.Cell

      `addPixel, equals, getArea, getAreaEnergy, getCellTypeColor, getCellTypeColor, getCellTypeColorName, getCellTypeFromID, getCenterOfMass, getComX, getComY, getConstants, getDisplay, getDistanceToCell, getID, getInformation, getInteractionEnergy, getIsoperimetricQuotient, getLocalAreaEnergy, getLocalPerimeterEnergy, getNeighborCells, getNotificationSource, getPerimeter, getPerimeterEnergy, getPixel, getShapeMetric, getType, hashCode, initalize, isIsolated, isIsolated, isMedium, removePixel, toString, updateParameters`
    - ### Methods inherited from class mvc.AObservable

      `addObserver, getNotificationSequenceNumber, notifyObservers, removeAllObservers, removeObserver`
    - ### Methods inherited from class java.lang.Object

      `equals, getClass, notify, notifyAll, wait, wait, wait`

- - ### Constructor Detail


    - #### SquareCell

      ```
      public SquareCell(Cell.CellType pType,
                        int pID,
                        Lattice pLattice)
      ```


    - #### SquareCell

      ```
      public SquareCell(int pID,
                        Lattice pLattice)
      ```


    - #### SquareCell

      ```
      public SquareCell(SquareCell pCell)
      ```


Skip navigation links


- Overview
- Package
- Class
- Use
- Tree
- Deprecated
- Index
- Help

- Prev Class
- Next Class

- Frames
- No Frames

- All Classes

- Summary:
- Nested |
- Field |
- Constr |
- Method

- Detail:
- Field |
- Constr |
- Method
